# Sprouts of plant ecophysiology along 100 years of *plant physiology*

**DOI:** 10.1093/plphys/kiag717

**Published:** 2026-09-24

**Authors:** Alicia V Perera-Castro, Jaume Flexas

**Affiliations:** Research Group on Plant Biology under Mediterranean Conditions, Universitat de les Illes Balears (UIB), Ctra Valldemossa Km 7.5, 07122 —Instituto de Investigaciones Agroambientales y de Economía del Agua (INAGEA), Carrer Blaise Pascal, 6, 07120, Palma, Illes Balears, Spain; Research Group on Plant Biology under Mediterranean Conditions, Universitat de les Illes Balears (UIB), Ctra Valldemossa Km 7.5, 07122 —Instituto de Investigaciones Agroambientales y de Economía del Agua (INAGEA), Carrer Blaise Pascal, 6, 07120, Palma, Illes Balears, Spain

## Abstract

Plant ecophysiology is the discipline within plant physiology that focuses on understanding how physiological processes in relation to environmental changes occur and how such responses differ among species. In this review, we provide a brief introduction to the historical corners that built the foundations of the discipline. Then, to celebrate the 100th anniversary of *Plant Physiology*, we highlight a sample of the key papers published by the journal over a century that have been landmarks in the development of Plant Ecophysiology as the well-structured discipline that it is nowadays. These are discussed within a broader historical and conceptual framework that synthesizes the development of 4 major research areas: plant growth and survival under environmental constraints, plant water relations and hydraulics, photosynthetic gas exchange, and photochemistry and photoprotection. By doing so, we aim to provide both a historical perspective and an assessment of the current state of the art across these interlinked domains of plant ecophysiology.

## Introduction

Plant ecophysiology is the branch of plant science that seeks to understand how physiological processes determine plant performance under natural environmental variability, integrating organismal function with field-based environmental complexity. Unlike strictly controlled laboratory physiology, ecophysiology is inherently rooted in measurements conducted under fluctuating conditions, where plants experience simultaneous variation in light, temperature, water availability, and atmospheric evaporative demand. This requires a combination of field experimentation, portable instrumentation, and targeted sampling for subsequent laboratory analyses. Typical variables include growth dynamics, stress tolerance indicators, gas exchange, hydraulic function, photochemical efficiency, and biochemical regulation. In this sense, modern ecophysiology spans several tightly connected domains: growth and survival under stress, water relations and plant hydraulics, photosynthetic gas exchange, and photochemistry and photoprotection. In the current “omics” era, its continued relevance lies in linking molecular-scale information with integrative, whole-plant function measured in situ (Flexas and Gago 2018). Besides these domains, which are those treated here based on the relevant papers published in *Plant Physiology* along a Century, there are many others, much less explored but for sure not less important, among which we highlight root responses to the environment, including abiotic (eg salt stress, water shortage, ion toxicities…) and biotic (eg mycorrhiza, soil microbiome…) conditions. From here we urge the *ecofizzers*' community to work to balance our current knowledge on the ecophysiology of the “hidden” below-soil part of plants to that of the most studied canopy.

While the roots of plant ecophysiology may be traced back to very early research, including works by Jan Baptiste van Helmont (16 to 17th centuries), Stephen Hales (17 to 18th centuries) or Alexander von Humoldt (18 to 19th centuries), its conceptual foundations were established between the late nineteenth century and the first half of the twentieth century and the term “plant ecophysiology” was coined by Heinrich Walter (“*ökophysiologischer*”) in 1964 (Walter 1964), although its major expansion occurred during the second half of the twentieth century, particularly in the 1970s and 1980s. Early contributions emerged from a diverse range of disciplines including plant geography, meteorology, and crop physiology, reflected in seminal works such as Blackman's formulation of limiting factors in growth (Blackman 1905), the ecological-genetic framework of plant adaptation (Turesson 1922), and early quantitative treatments of environmental control of plant production (Monsi and Saeki 1953; Gates 1962). During this period, physiological ecology began to consolidate as a distinct discipline, as synthesized by Billings (1957), while integrative modeling approaches to plant growth and resource allocation were developed by authors such as Brouwer and de Wit (1969). These foundations were progressively expanded into a systems-oriented view of plant function, incorporating stomatal regulation (Cowan and Farquhar 1977), plant-water relations (Slatyer 1967), and ecosystem-level productivity constraints (Monteith 1972), establishing the conceptual basis for modern ecophysiology.

The subsequent “bloom” of ecophysiology between 1970 and 2000 was characterized by an unprecedented expansion of field-based plant physiological research, with a strong presence in multidisciplinary journals such as *Nature* and *Science*, alongside a remarkable body of work published in specialized journals. In this sense, these 2 journals served as key outlets for field-based ecophysiological breakthroughs, including studies on photosynthetic adaptation to environmental stress (Berry 1975), carbon isotope ecology (Troughton et al. 1974), and physiological ecology in extreme environments (Mooney 1972; Ehleringer et al. 1976). This reflects a period in which ecophysiology was strongly field-driven and often disseminated through broadly interdisciplinary journals rather than specialized physiological outlets.

Among specialized journals, although *Plant Physiology* was widely regarded as one of the discipline's leading physiological journals, surprisingly quite some of the most influential conceptual advances in ecophysiology appeared elsewhere, particularly in *Planta*, which hosted several landmark contributions including fundamental analyses of leaf energy balance and transpiration (Raschke 1956), stomatal regulation under desert conditions (Schulze et al. 1972)—which later led to a deep understanding of the complexity of factors involved in stomatal regulation, as well as 2 of the major landmarks ever in ecophysiology of photosynthesis, ie the biochemical framework of photosynthesis (Farquhar et al. 1980; von Caemmerer and Farquhar 1981) and the chlorophyll fluorescence equivalences for quantum yield of photosynthesis (Björkman and Demmig 1987; Demmig and Björkman 1987).

Despite the apparent historical predominance of other journals in the development of plant ecophysiology, *Plant Physiology* has consistently published a series of influential contributions that have shaped key aspects of the discipline. Even for our own research, *Plant Physiology* has long been a place where we have aimed to publish our best work, sometimes successfully (Flexas et al. 1999; Gago et al. 2016). Precisely in relation to mesophyll conductance, 2 of the most influential contributions published in *Plant Physiology* were those of Harley et al. (1992), who highlighted the importance of internal CO_2_ diffusion in interpreting gas exchange measurements, and Bernacchi et al. (2002), who incorporated mesophyll conductance into the estimation of in vivo Rubisco kinetics and refined the interpretation of photosynthetic limitations in terms of chloroplastic CO_2_ concentration.

In a broader context of carbon assimilation efficiency and crop productivity under environmental change, several studies published in *Plant Physiology* have provided key insights into the functioning of photosynthesis at canopy and ecosystem scales. Leakey et al. (2006) showed that elevated atmospheric CO_2_ does not necessarily enhance maize productivity under nonwater-limited conditions. Zhu et al. (2007) used computational optimization approaches to demonstrate that photosynthetic efficiency could be substantially improved through reallocation of enzymatic resources within carbon metabolism.

In parallel, major advances in plant hydraulic and stomatal physiology have been documented. Brodribb and Holbrook (2003) linked stomatal closure during dehydration to leaf physiological traits associated with water status regulation. Brodribb et al. (2007) demonstrated a tight coordination between maximum photosynthetic capacity and leaf venation architecture across major plant lineages, emphasizing hydraulic constraints on carbon gain. Brodribb and Cochard (2009) showed that hydraulic failure defines both recovery capacity and mortality thresholds under severe drought stress in conifers. McAdam and Brodribb (2015, 2016) explored the evolutionary and hormonal basis of stomatal responses to vapor pressure deficit across land plants and their mechanisms in cell turgor and volume (see also Sack et al. 2018), while Torres-Ruiz et al. (2015) provided direct visual evidence of xylem embolism formation under tension using X-ray microtomography. Complementing these observations, Scoffoni et al. (2017, 2018) showed that hydraulic decline in leaves during dehydration is strongly controlled by outside-xylem pathways, preceding or occurring independently of xylem embolism, and provided the first direct visualization of embolism formation in leaves. More recently, Pichaco et al. (2024) demonstrated that stomatal opening kinetics are mechanically constrained by evaporative demand, providing new insights into dynamic stomatal behavior under fluctuating environments.

Finally, photoprotective and energy dissipation mechanisms under combined light and water stress have also been addressed within *Plant Physiology*. Demmig-Adams et al. (1989a, 1989b) showed that the xanthophyll cycle plays a central role in the thermal dissipation of excess excitation energy under conditions of high irradiance, drought and limited availability of Rubisco substrates, linking photochemistry with stress physiology. Later, Murchie and Niyogi (2011) provided a comprehensive review of the physiological principles underlying photoprotection and photoinhibition, while also questioning whether these processes are optimized in terms of carbon gain.

To address this review, we were kindly provided by the journal a list containing the 5 most cited papers per decade over the past 100 yr, together with the 3 most downloaded articles and the 2 highest-ranked papers in terms of Altmetric attention (ie a total of 10 highlighted paper per decade). From this dataset, we selected the contributions more closely related to plant ecophysiology (Table 1). These are discussed within a broader historical and conceptual framework that synthesizes the development of 4 major research areas: plant growth and survival under environmental constraints, plant water relations and hydraulics, photosynthetic gas exchange, and photochemistry and photoprotection. By doing so, we aim to provide both a historical perspective and an assessment of the current state of the art across these interlinked domains of plant ecophysiology. We recognize that this approach does not provide a comprehensive history of plant ecophysiology, as many important contributions were published in other journals and measures such as citation, download, and Altmetric counts do not necessarily capture scientific impact or historical influence. Rather, our selection provides a perspective on how major themes in plant ecophysiology have been represented within the pages of *Plant Physiology* over the past century.

**Table 1:** Selected contributions to plant ecophysiology among the most cited papers of *Plant Physiology* over the past 100 yr.

Reference	Title	Contribution	All time citations^a^	Reason for selection
*Environmental controls of plant growth and survival*				
Veihmeyer and Hendrickson (1927)	Soil-moisture conditions in relation to plant growth	Established the relationship between soil moisture availability and plant growth, providing a foundation for understanding plant responses to water deficit.	92	Top download
Dexter et al. (1932)	Investigations of the hardiness of plants by measurement of electrical conductivity	Pioneered the use of electrolyte leakage, measured as electrical conductivity, as a quantitative indicator of plant freezing injury and cold hardiness.	248	Top cited
Singh and Lal (1935)	Limitations of Blackman's law of limiting factors and Harder's concept of relative minimum as applied to photosynthesis	Provided compelling evidence that photosynthesis is controlled by the combined effects of multiple environmental factors, challenging the strict application of Blackman's law of limiting factors.	28	Top download
Nelson (1944)	Growth responses of hemp to differential soil and air temperatures	Demonstrated that plant growth is strongly influenced by the independent effects of soil and air temperatures, highlighting the importance of root-zone temperature in plant development and stress responses.	15	Top Altmetric
Haagen-Smit et al. (1952)	Investigation on injury to plants from air pollution in the Los Angeles area	Established plants as bioindicators of air pollution by characterizing visible foliar injury caused by photochemical smog, laying the foundation for studies of pollution-induced plant stress.	155	Top Altmetric
Boese and Huner (1990)	Effect of growth temperature and temperature shifts on spinach leaf morphology and photosynthesis	Showed that cold acclimation enhances resistance to low temperature-induced photoinhibition without reducing photosynthetic efficiency, emphasizing the role of thermal acclimation in plant cold tolerance	124	Top download
*Gas exchange in photosynthesis*				
Myers and Kratz (1955)	Photosynthesis and respiration of 3 blue-green algae	Provided one of the first quantitative analyses of photosynthesis and respiration in cyanobacteria, establishing the basis for studies of carbon metabolism in photosynthetic microorganisms.	92	Top Altmetric
Hew et al (1969)	Effects of temperature on photosynthesis and CO_2_ evolution in light and darkness by green leaves	Showed that temperature differentially affects CO_2_ evolution in light and darkness, highlighting the temperature dependence of carbon metabolism in plants.	52	Top Altmetric
Ku et al. (1977)	Effects of light, carbon dioxide, and temperature on photosynthesis, oxygen inhibition of photosynthesis, and transpiration in *Solanum tuberosum*	Demonstrated the interactive effects of light, CO_2_, and temperature on photosynthesis, photorespiration, and transpiration, establishing the environmental regulation of photosynthetic performance in potato.	143	Top download
Koch and Kennedy (1982)	Crassulacean acid metabolism in the succulent C_4_ dicot, *Portulaca oleracea* L under natural environmental conditions	Showed that Portulaca oleracea can induce CAM under natural conditions, highlighting the plasticity of photosynthetic metabolism in response to environmental stress.	58	Top Altmetric
Harley et al (1992)	Theoretical considerations when estimating the mesophyll conductance to CO_2_ flux by analysis of the response of photosynthesis to CO_2_	Provided the theoretical basis for estimating mesophyll conductance from photosynthetic CO_2_ response analyses, enabling more accurate assessment of internal limitations to photosynthesis.	902	Top cited
*Light absorption and photoprotection*				
Moss and Loomis (1952)	Absorption spectra of leaves. I. The visible spectrum	Demonstrated the role of leaf structure in light absorption together with chlorophylls and carotenoids.	127	Top download
Woolley (1971)	Reflectance and transmittance of light by leaves	Characterized leaf reflectance and transmittance, laying the foundation for optical sensing and remote assessment of vegetation.	585	Top download
Demmig et al (1987)	Photoinhibition and zeaxanthin formation in intact leaves: A possible role of the xanthophyll cycle in the dissipation of excess light energy	Demonstrated the close relationship between zeaxanthin formation and photoprotection during photoinhibition, identifying the xanthophyll cycle as a key mechanism for dissipating excess light energy.	739	Top Altmetric
Loreto and Velikova (2001)	Isoprene produced by leaves protects the photosynthetic apparatus against ozone damage, quenches ozone products, and reduces lipid peroxidation of cellular membranes	Demonstrated that isoprene emission protects the photosynthetic apparatus against ozone-induced oxidative damage by scavenging reactive oxidation products and reducing membrane lipid peroxidation.	1,257	Top cited
*Plant water relations and hydraulics*				
Scholander et al. (1962)	Salt balance in mangroves	Demonstrated the mechanisms by which mangroves maintain salt balance, establishing the physiological basis of salt tolerance in halophytic plants.	221	Top download
Marshall (1958)	Measurement of sap flow in conifers by heat transport	Pioneered the use of heat transport to measure sap flow, establishing a nondestructive method for quantifying plant water transport.	433	Top cited
Boyer (1968)	Relationship of water potential to growth of leaves	Demonstrated the relationship between leaf water potential and growth, establishing water potential as a key determinant of plant growth under water deficit.	292	Top download
Smart and Bingham (1974)	Rapid estimates of RWC	Introduced a rapid method for estimating RWC, providing a simple and reliable indicator of plant water status.	785	Top cited
Brodribb and Holbrook (2003)	Stomatal closure during leaf dehydration, correlation with other leaf physiological traits	Established the relationship between stomatal closure and leaf hydraulic traits during dehydration, advancing the understanding of hydraulic regulation of drought responses.	693	Top download
McDowell (2011)	Mechanisms linking drought, hydraulics, carbon metabolism, and vegetation mortality	Established a conceptual framework linking drought, hydraulic failure, carbon starvation, and vegetation mortality.	1,155	Top cited

^a^The number of citations is not intended to serve as a ranking of the quality of the listed papers, but rather as a measure of the impact the work has had on the scientific community, which is clearly influenced by the number of researchers working in the same field of research. Current citations are those retrieved from the Dimensions database as consulted on 10th July 2026.

### Environmental controls of plant growth and survival

Research on environmental controls of plant growth and survival emerged from early efforts to understand how environmental factors constrain plant performance, following the conceptual framework established by Blackman's law of limiting factors (Blackman 1905). Within this context, early experimental work in *Plant Physiology* already reflected a shift toward quantitative plant-environment relationships, as exemplified by early studies such as those by Veihmeyer and Hendrickson (1927) and Singh and Lal (1935), which contributed to the development of controlled physiological experimentation on growth responses under environmental variation. By that time, Dexter et al. (1932) introduced electrolyte leakage measurements as a quantitative indicator of freezing injury, providing one of the earliest physiological approaches to assessing plant stress tolerance, a methodology that remains widely used today. Nelson (1944) demonstrated the importance of soil temperature as a determinant of plant growth, highlighting the integrated influence of above- and below-ground environments. It is noteworthy—and still relevant—that more than 80 yr later, many experimental studies continue to overlook or underreport soil temperature, despite its potential importance in processes such as drought responses, particularly in pot experiments. Expanding the range of environmental constraints considered by plant physiologists, Haagen-Smit et al. (1952) identified atmospheric pollutants as direct causes of plant injury, laying the foundations for later studies of anthropogenic stress. These developments contributed to the broader emergence of plant stress physiology, synthesized in the influential work of Levitt (1972). By the late twentieth century, attention had shifted from injury assessment to mechanisms of acclimation, culminating in studies such as Boese and Huner (1990), which linked growth temperature to coordinated changes in leaf morphology and photosynthetic performance, building on the conceptual framework of thermal acclimation developed by Berry and Björkman (1980).

### Gas exchange in photosynthesis

The history of gas exchange and photosynthesis research mirrors the broader development of plant ecophysiology, from early measurements of carbon assimilation to mechanistic models linking leaf structure, biochemistry, and environmental responses. Following the pioneering demonstrations of photosynthetic carbon fixation by Blackman (1905) and the elucidation of the photosynthetic carbon reduction cycle by Calvin and colleagues (Bassham et al. 1950), quantitative gas-exchange approaches became central to plant physiology. Among the earliest contributions in *Plant Physiology*, Myers and Kratz (1955) characterized photosynthetic and respiratory processes in cyanobacteria, extending photosynthetic research beyond higher plants and helping establish photosynthetic microorganisms as experimental systems. Interest subsequently shifted toward environmental controls of carbon assimilation, exemplified by Hew et al. (1969), who analyzed the temperature dependence of photosynthesis and respiration, anticipating later studies of thermal acclimation. During the 1970s, advances in biochemical and ecological perspectives converged through the discovery of alternative photosynthetic pathways. Kortschak et al. (1965) provided an early indication of the distinctive CO_2_ fixation pathway in sugarcane by showing that malate and aspartate were the first stable products of carbon fixation, paving the way for the identification of the C_4_ pathway by Hatch and Slack (1966). This biochemical framework, together with the ecological analyses of Björkman (1968), contributed to understand the environmental regulation and adaptative significance of photosynthetic pathways. Within this context, Koch and Kennedy (1982) demonstrated the operation of CAM under natural conditions in *Portulaca oleracea*, highlighting the ecological diversity of photosynthetic pathways and their adaptive significance in water-limited environments. On the other hand, Ku et al. (1977) examined the interactive effects of light, CO_2_, temperature, and oxygen on photosynthesis and transpiration, providing experimental evidence for several of the environmental controls that would later be incorporated into mechanistic photosynthesis models. These developments culminated in the landmark biochemical model of C_3_ photosynthesis proposed by Farquhar, von Caemmerer and Berry (1980). The subsequent integration of gas-exchange measurements with carbon isotope discrimination theory (Farquhar et al. 1982), stomatal regulation (Cowan and Farquhar 1977), and biochemical limitations of photosynthesis (von Caemmerer and Farquhar 1981) transformed photosynthesis research into a predictive framework applicable across scales. A major contribution from *Plant Physiology* was provided by Harley et al. (1992), whose theoretical analysis of mesophyll conductance helped establish internal CO_2_ diffusion as a critical determinant of photosynthetic performance. This work paved the way for a new generation of studies integrating diffusional and biochemical limitations to photosynthesis. Particularly influential were the temperature-dependent kinetic parameterizations of Rubisco developed by Bernacchi et al. (2001), which refined the application of the Farquhar model to A–Ci analyses, and the subsequent incorporation of chloroplastic CO_2_ concentrations into photosynthetic modeling (Bernacchi et al. 2002). Together with growing evidence for the ecological significance of mesophyll conductance under drought and other environmental stresses (Flexas et al. 2008), these advances transformed the interpretation of gas-exchange measurements and established the framework for modern analyses of photosynthetic limitations. More recent studies, including many of our own research group, further linked mesophyll conductance with leaf metabolism and anatomy (Flexas et al. 2006, 2021; Gago et al. 2016, 2019; Clemente-Moreno et al. 2019; Nadal et al. 2021; Carriquí et al. 2019; Perera-Castro et al. 2022), providing a mechanistic basis for variation in photosynthetic capacity across species and environments.

Together, these studies illustrate the progression from descriptive measurements of gas exchange to an integrated understanding of photosynthesis as the outcome of coordinated biochemical, diffusional, and environmental constraints.

### Light absorption and photoprotection

In parallel with advances in carbon assimilation, pioneering studies began to provide an experimental foundation for understanding photosynthetic efficiency and the relationship between pigment composition and light utilization in plants. Several of these landmark studies were published in *Plant Physiology*. Moss and Loomis (1952) analyzed the absorption spectra of leaves from different species in the visible region and compared them with those of pigment extracts, demonstrating that leaf structure (through light scattering and reflection) modifies the observed absorption relative to isolated pigments, while confirming the central role of chlorophylls and carotenoids in light absorption. Later, Woolley (1971) further investigated the spectral behavior of leaves. He showed that the interfaces between cells and intercellular air spaces produce strong light scattering and that leaf water content substantially influences spectral responses, particularly in the near-infrared region. These findings laid the foundation for the development—decades ago—of vegetation remote sensing and spectral analysis techniques.

Beyond the optical properties of leaves, by the mid-1980s the existence of the xanthophyll cycle and its biochemical regulation had been firmly established (Sapozhnikov et al. 1957; Hager 1957, 1967; Yamamoto et al. 1962; Hager and Stransky 1970), although its physiological function remained unclear. A major breakthrough came with the work of Demmig et al. (1987), which provided some of the first experimental evidence that zeaxanthin, produced through the xanthophyll cycle, plays a photoprotective role by dissipating excess light energy absorbed by leaves. Although this hypothesis was initially presented with caution, it was later confirmed by genetic and molecular studies in the 1990s (Niyogi et al. 1998). Another major contribution of Barbara Demmig-Adams and her group was the development of the concept of zeaxanthin-dependent nonphotochemical quenching. This concept was further strengthened by Demmig et al. (1988), who demonstrated that the same photoprotective mechanism operates under physiologically relevant environmental stress conditions, specifically the combination of high irradiance and water deficit. Another important contribution of this work was the use of pulse-amplitude modulated (PAM) chlorophyll fluorometry. This technique had been developed by Uli Schreiber et al. (1986) and was subsequently linked to the actual efficiency of PSII photochemistry under light conditions by Genty et al. (1989). The application of PAM chlorophyll fluorometry by Demmig and co-workers to quantify energy dissipation and relate it to changes in carotenoid composition established an experimental approach that has since become widely adopted in photoprotection research.

Building on this framework, Loreto and Velikova (2001) provided some of the first experimental evidence that isoprene functions as a defense mechanism against oxidative stress. They showed that leaves emitting isoprene in response to ozone treatment exhibited significantly lower energy dissipation than leaves in which isoprene biosynthesis had been inhibited, while maintaining higher photosynthetic performance. This study naturally complemented the zeaxanthin-based photoprotection model developed during the previous decade. Whereas zeaxanthin prevents the formation of reactive oxygen species (ROS) by dissipating excess energy excitation before it reaches damaging levels, isoprene acts primarily after oxidative stress has been initiated, scavenging reactive oxidants and limiting damage to cellular membranes.

### Plant water relations and hydraulics

In addition to the advances in photosynthesis research, *Plant Physiology* also played a central role in shaping our understanding of plant water relations. Early methodological breakthroughs provided the foundation for quantifying plant water status and its impact on physiological processes. Marshall (1958) developed one of the first methods for estimating sap velocity in intact conifers. Inspired by earlier studies on heat conduction by Huber and Schmidt (1937) and Dixon (1937), the method involved applying a heat source to the trunk and measuring how the resulting heat was transported upward by the flow of water through the xylem. Sap velocity estimated using Marshall's physical model of heat transport closely matched the expected transpiration responses of conifers under varying environmental conditions, including changes in radiation, temperature, and atmospheric evaporative demand. More accurate techniques for measuring sap flow were subsequently developed, including the heat pulse velocity method, Granier's thermal dissipation probes, and heat balance methods. Nevertheless, all these approaches are conceptually rooted in the physical framework introduced by Marshall.

A comparable breakthrough was the development of the pressure chamber (pressure bomb) technique (Scholander et al. 1964), first applied to mangroves, which provided a rapid and reliable method for estimating leaf and stem water potential and soon became one of the most widely used tools in plant ecophysiology. This work was preceded by an earlier study from the same research group published in *Plant Physiology*, which experimentally demonstrated that mangroves are primarily “salt excluders” rather than simply “salt secretors” (Scholander et al. 1962). The xylem sap of the species examined contained salt concentrations far lower than those of seawater, a difference that could not be explained by salt secretion through leaf glands alone. The authors therefore concluded that mangrove survival depends primarily on restricting salt entry at the roots rather than excreting it after uptake. At the same time, the leaves maintained highly negative osmotic potential through the accumulation of compatible solutes, allowing continued water uptake from saline soils. Together, these studies laid the foundation for the modern ecophysiology of halophytic plants.

Analogous to the widespread adoption of the pressure chamber pioneered by Scholander and colleagues, the use of thermocouple psychrometers became increasingly popular following Boyer's (1968) studies on leaf growth responses to water deficit, who demonstrated that relatively small reductions in leaf water potential were sufficient to markedly inhibit leaf expansion, establishing plant water status as a primary regulator of growth. During the growth phase, the leaves maintained a relatively constant water potential, approximately between −1.5 and −2.5 bar, even when their petioles were immersed in pure water. This finding demonstrated that a growing leaf never reaches hydraulic equilibrium with the available water because a water potential gradient must be maintained to drive water into expanding cells.

Shortly thereafter, Smart and Bingham (1974) introduced a rapid method for estimating plant water status that, unlike the conventional determination of relative water content (RWC), did not require measurement of dry weight. They showed that, for a given species, the Relative Tissue Weight (RTW, defined as the ratio of fresh weight to turgid weight) exhibited an almost perfectly linear relationship with RWC. Although an initial calibration was still required for each species, it could be performed using only a small number of subsamples. This greatly reduced the time required for analysis and enabled much larger sample sizes, thereby facilitating physiological and agronomic studies of plant responses to water stress. More than 50 yr after its publication, the method continues to be included in experimental protocols and plant physiology manuals, making this short paper one of the most influential methodological contributions published in *Plant Physiology*.

Several decades later, the integration of hydraulic and physiological approaches transformed the field. Brodribb and Holbrook (2003) demonstrated that declines in leaf hydraulic conductance occur in parallel with reductions in stomatal conductance, providing compelling evidence that leaf hydraulic properties play a key role in regulating gas exchange during drought. The turgor loss point closely coincided with substantial stomatal closure, whereas declines in photochemical efficiency occurred only at much more negative water potentials. These findings indicated that stomatal closure is primarily a hydraulic response aimed at protecting the leaf water transport system, rather than a direct consequence of impaired photosynthesis, thereby establishing plant hydraulics as a central framework for understanding drought responses. Building on this hydraulic perspective, McDowell (2011) published a conceptual synthesis that redefined one of the central debates in modern plant ecophysiology: what ultimately causes plants to die during drought? Until then, the field had largely been polarized between 2 competing hypotheses: (ⅰ) hydraulic failure, whereby mortality results from the loss of xylem water transport capacity due to cavitation and embolism, and (ⅱ) carbon starvation, whereby prolonged stomatal closure limits photosynthesis to such an extent that carbon reserves become depleted. McDowell's major contribution was to argue that these mechanisms are not mutually exclusive but are tightly coupled, with the relative importance of each depending on drought duration and severity, as well as species-specific physiological responses. In this framework, stomatal closure represents a physiological tradeoff: it protects the hydraulic system while simultaneously increasing the risk of carbon limitation. Furthermore, McDowell highlighted that assessing carbon starvation requires more than simply quantifying nonstructural carbohydrate (NSC) reserves. It is equally important to determine whether those reserves remain physiologically accessible. A plant may retain substantial amounts of sugars and starch while nevertheless experiencing functional carbon limitation because drought impairs phloem transport and causes loss of cellular turgor, preventing carbohydrates from being mobilized to the tissues where they are required. This conceptual framework has profoundly influenced contemporary research on forest responses to climate change and continues to guide studies of plant resilience under increasingly frequent and severe drought events.

### Final statement

Looking back at these milestones illustrates that conceptual advances arise not from new technologies alone, but from their ability to answer fundamental physiological questions in an ecological context. As plant science enters an increasingly omics-driven era, maintaining a strong ecophysiological framework will be essential for translating molecular information into predictive understanding of plant responses to a changing environment. Molecular technologies can identify genes, pathways, and regulatory networks, but they do not replace ecophysiology, which can determine how these translate into whole-plant function and ultimately shape plant performance in natural environments. We should definitely enter ‒and indeed we are progressively entering‒ an “ecophysiolomics era”, a concept proposed less than a decade ago by Flexas and Gago (2018) to describe the integration of omics approaches with ecophysiology, linking comprehensive molecular, physiological, and environmental information to understand and predict plant performance under natural and changing environments.

## Data Availability

There are no data associated with this review beyond the list of references presented in Table 1.
